# Using the Think-Aloud Method to Assess the Feasibility and Acceptability of Network Canvas Among Black Men Who Have Sex With Men and Transgender Persons: Qualitative Analysis

**DOI:** 10.2196/30237

**Published:** 2021-09-09

**Authors:** Natalie D Crawford, Dorie Josma, Kristin R V Harrington, Joseph Morris, Alvan Quamina, Michelle Birkett, Gregory Phillips II

**Affiliations:** 1 Department of Behavioral, Social, and Health Education Sciences Rollins School of Public Health Emory University Atlanta, GA United States; 2 Department of Epidemiology Rollins School of Public Health Emory University Atlanta, GA United States; 3 Department of Population Health School of Public Health Georgia State University Atlanta, GA United States; 4 NAESM Inc Atlanta, GA United States; 5 Department of Medical Social Sciences Feinberg School of Medicine Northwestern University Chicago, IL United States

**Keywords:** think-aloud, egocentric networks, sociogram, social networks, MSM, transgender, network canvas, black MSM, infectious disease transmission, stigma

## Abstract

**Background:**

Characteristics of an individual’s social network have been important factors in understanding infectious disease transmission patterns. Social network data collection is generally time and resource intensive, yet it is crucial to our understanding of the complex epidemiologic landscape of human behaviors among stigmatized social groups.

**Objective:**

We sought to evaluate the feasibility and acceptability of a self-administered social network data collection tool, Network Canvas, among Black men who have sex with men (BMSM) and transgender persons using the think-aloud method, which is a robust and flexible research technique used to perform usability testing.

**Methods:**

We piloted a self-administered network interview within the Network Canvas Software Suite. Participants aged 18 years and older were recruited through a community-based organization in Atlanta, GA, and were included based upon their willingness to share information on sexual behaviors and drug use for themselves and their social networks. A semistructured interview guide was used to document cognitive decision-making processes while using the tool. Recorded interviews were transcribed verbatim, and thematic analyses were performed.

**Results:**

Among 7 BMSM and transgender participants, three main themes were identified from cognitive processes: (1) the utility, (2) navigation, and (3) intuitive design of Network Canvas. Overall, Network Canvas was described as “easy to use,” with suggestions mainly directed toward improving navigation tools and implementing an initial tutorial on the program prior to use. Participants were willing to use Network Canvas to document their social networks and characteristics. In general, observed verbal responses from participants matched their behavior, although there were some discrepancies between verbal affirmations of use and understanding versus external observation.

**Conclusions:**

We found Network Canvas to be a useful new tool to capture social network data. Self-administration allowed participants the opportunity to provide sensitive information about themselves and their social networks. Furthermore, automated name generation and visualization of an individuals’ social network in the app has the potential to reduce cognitive burden during data collection. More efficient methods of social network data collection have the potential to provide epidemiologic information to guide prevention efforts for populations with stigmatized health conditions or behaviors.

## Introduction

Social networks are understood as patterns of stable interactions among people [1,2] that can be categorized as instrumental, supportive, disruptive, burdensome, or neutral. Infectious disease transmission, such as HIV transmission, requires interactions between at least two individuals. Thus, characteristics of an individual’s social network have been important factors in understanding infectious disease transmission patterns. Indeed, various social network characteristics have been identified as robust predictors of HIV transmission. For example, an individual’s network size, demographics of the network, and the individual’s position in their own network are highly linked to the risk of sexually transmitted infections [3], and sexual [4-6] and substance use [7-10] behaviors.

The ability to capture valid reports of social network data is crucial in order to assess the relationships between social networks and HIV. Social network inventories have mainly been collected using standard data collection methods that ask research participants to list people within their networks during a specified period and then to provide potentially extensive information about each alter’s demographics, perceived behaviors, and health outcomes [11]. Although the use of paper social network inventories is effective and has produced reliable reports even with historical accounts of social networks, the data collection, entry and cleaning processes for these data are cumbersome for both participants and researchers [11]. Moreover, the fatigue related to this method of social network data collection may lead to underreporting of network members and data entry errors [12,13]. Electronic social network data collection platforms that circumvent some of these issues have recently emerged. For example, electronic social network data collection platforms can reduce survey length by providing electronic data entries instead of written data entries, drag and drop features to report network characteristics, and backend programming that stores data into a ready-to-analyze format with several automated network characteristic measures such as network size [14,15].

Network Canvas is a recently developed, open-source, electronic social network data collection platform that is intuitively designed to collect and export social network data [16,17]. As described previously [18], Network Canvas aims to simplify the collection and management of network data via the use of touch-optimized interfaces for data capture. Through these features, researchers can assess more nuanced associations between contextual factors and infectious disease spread, and they are able to use these data in near real-time. Network Canvas has been extensively tested to ensure that it is not only user friendly but also effective and efficient before its stable release. Previous evaluations of Network Canvas were completed on a sample of young men who have sex with men (MSM) to understand their social, drug use, and sexual networks. Researchers found that Network Canvas maintained data quality comparable to other digital platforms, and young MSM found Network Canvas easy to use [17,19]. However, the utilization of Network Canvas is still limited, and the platform was designed to be an interviewer-assisted platform. Although such platforms can be useful in certain settings, the lack of validation of it as a self-administered tool may limit the utility of Network Canvas in some research and clinical settings with fewer resources and limit its use with some of the most in-need populations.

Limited resources, including fewer staff, less time, and budgetary restrictions, are common in research and clinical settings that serve racial minority MSM, who have the highest risk of HIV transmission [20,21]. Thus, research is needed to determine whether Network Canvas’ streamlined and intuitive design can be self-administered in a community setting. The purpose of this study is to evaluate the feasibility and acceptability of a self-administered social network data collection inventory on Network Canvas among Black MSM (BMSM) and transgender persons using the think-aloud method. The think-aloud method is a robust and flexible research technique used to perform usability testing [22]. It allows participants to provide valuable, reliable, and unfiltered information of their cognitive process while completing a task [22,23]. The think-aloud method is widely used in the disciplines of psychology [23], engineering [24,25], education [22,26], and public health [27,28]. In public health, think-aloud methods have been used to assess participants’ cognitive understanding of novel survey measures. To our knowledge, this method has not been used in health research to understand whether study participants can adequately self-administer an electronic social network data collection tool by understanding the natural flow and expectations of the information being requested by the program. In this article, we utilized the think-aloud method to understand BMSM and transgender persons’ cognitive processes and then assessed the feasibility and acceptability of Network Canvas for personal social network data collection.

## Methods

### Design

Northwestern University partnered with researchers at the Rollins School of Public Health at Emory University to pilot the Network Canvas software app. In order to assess the feasibility and acceptability of using Network Canvas among BMSM and transgender persons, a semistructured interview guide was used to instruct participants in how to verbalize their cognitive decision-making process in real-time while completing a task or using a tool on Network Canvas. All study activities were approved by the Emory University Institutional Review Board.

### Recruitment

A total of 7 participants (N=7) were typically purposively recruited in collaboration with a community-based organization in Atlanta, GA. To be included in the study, all participants had to be aged 18 years or older and willing to share personal information on sexual behaviors and drug use for themselves and their social networks. Participant ages ranged between 19 and 28 years old. Participants self-identified as BMSM (n=5), transmen (n=1), and transwomen (n=1).

### Data Collection and Analysis

Data for this study were collected as a part of a parent study in which participants were interviewed in order to evaluate their willingness to receive sexual health services in pharmacies. Participants were subsequently asked about their willingness to participate in the feasibility and acceptability testing of Network Canvas. Prior to data collection, researchers downloaded the Network Canvas Architect Application, the component of the Network Canvas Software Suite that deploys study protocols to a password-protected iPad. Architect software (version 4.0.0; Complex Data Collective) was used.

**Figure 1:**
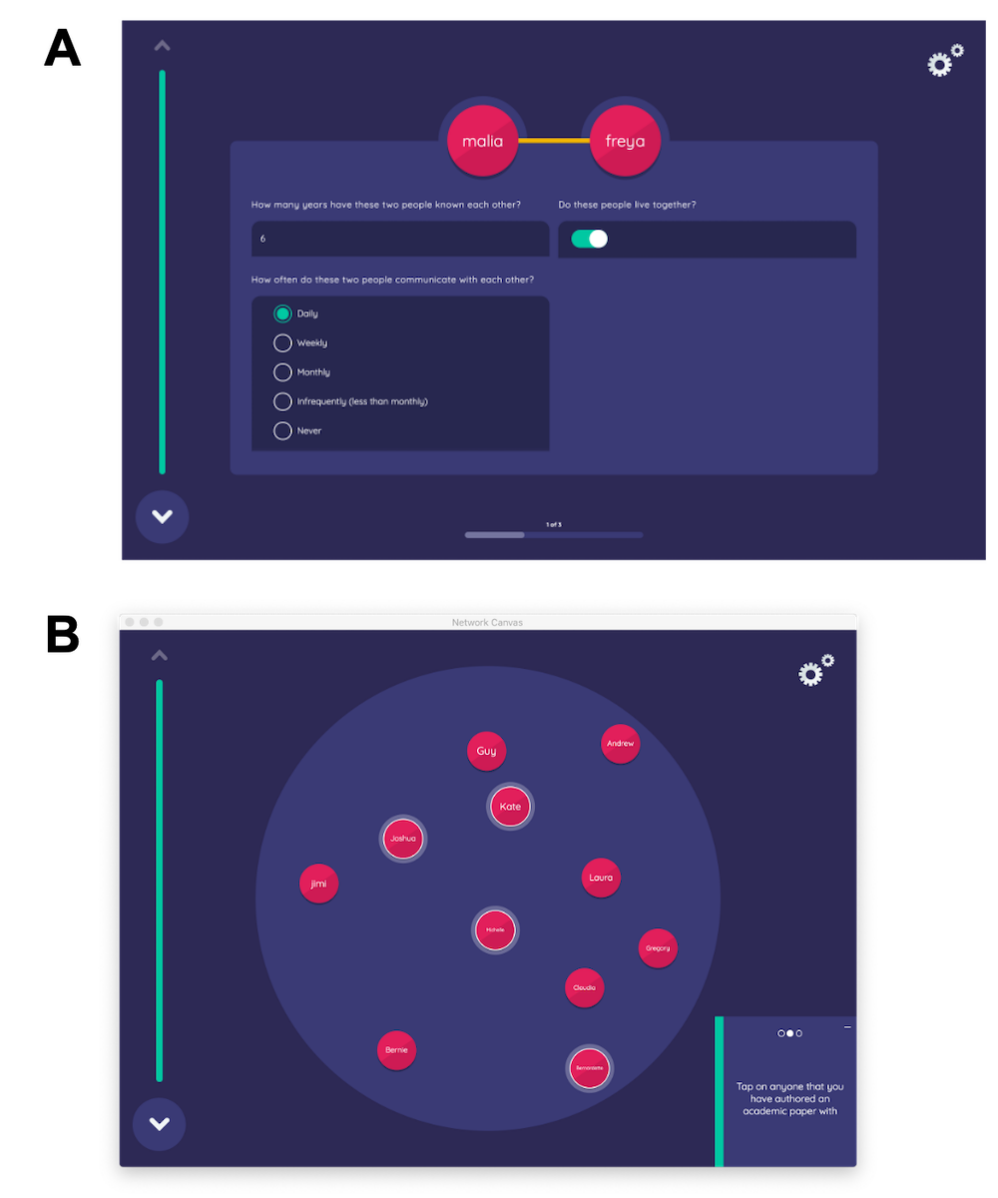
Screenshots of the Network Canvas interface and workflow. (A) Detailed edge interpretation for individual ties; (B) elicitation of ties within a sociogram.

In the Network Canvas protocol, participants were asked to identify their social networks and engagements with these networks in the last 3 months (see Figure 1 for example screenshots). In the name generator, participants assigned fictitious names to people in their social networks; these names were then linked to their reported demographic information (ie, age and sex), sexual health status, and drug use behaviors (ie, cocaine, heroin, or opioids). Participants were asked questions such as, “Who did you get together to hang out with or socialize?” “Who did you have sex with?” and “Who did you use drugs with?” Participants were also instructed to place each network on a sociogram. The names of individuals who were closest socially to the participants were arranged in the center of the circle, and those who were the least socially close to participants were placed on the margins of the circle.

Researchers used think-aloud methods in order to guide participants to verbally describe their cognitive process by using the features and tools while completing the Network Canvas social network inventory. Participants were asked to actively describe their understanding of the software program as they navigated it to complete the survey questions. Following the interview, participants were asked questions about their experience using the software such as, “Did you find the application intuitive?” and “How was your experience using the Network Canvas application to answer these questions?” All interviews were recorded using an audio-recorder. Researchers took observational notes to capture nonverbal cues not captured on the audio recorder. All data were uploaded to a secure computer server to maintain participants’ confidentiality. Each participant received a US $50 gift card to compensate for their time upon interview completion.

Research assistants with a masters-level training in qualitative methods transcribed the audio-recorded interviews verbatim. Participants’ verbatim transcripts were complemented with their respective observational notes to enhance the analysis of both verbal and nonverbal cues. NVivo 12 (Version 12; QSR International), a qualitative data analysis software, was used to perform a thematic analysis of the participants’ interviews and observation notes. Three transcripts were analyzed and used to develop a codebook with inductive codes, definitions, and in-text examples. The codebook was then used to code the remaining transcripts and generate findings. Saturation was determined when new emergent themes were no longer present in the data. Once saturation was reached, participant recruitment was stopped.

## Results

Cognitive processes of participants were captured while navigating through the Network Canvas application, and close attention was paid towards participants’ processes using the application’s features and tools. Analyzed data were organized into three main themes: (1) the utility, (2) navigation, and (3) intuitive design of Network Canvas.

### Utility of Network Canvas

All participants described Network Canvas as “easy to use.” They also shared that features within the application were useful in collecting and organizing personal network information. Specifically, one participant explained that the name generator was useful in allowing names to be added and populated on the sociogram for later use.

It was easy. It was very easy.

Participant #107

It’s an easy process and you don’t have to add them [names of social networks members] again. Just a simple tap.

Participant #112

### Navigation of Network Canvas

Although participants responded positively to the utility of the application, most of them expressed ways in which to improve navigation. The name generator and sociogram required participants to perform different data input tasks either by clicking, tapping, or dragging. Participants (6/7, 85.7%) found that these various tools between pages made it harder to engage with the application. During the interviews, participants asked the interviewers how to navigate between questions and pages.

So, it’s asking me a question but how am I supposed to—click this button here to answer…So, I guess I hit this arrow to move forward?... So, you’re saying if they’re highlighted, it means selected?

Participant #108

I’m going to the next question. Do I just click on them?... Okay so this person name is here, how do I get to the other person name?

Participant #109

The tapping and dropping and dragging…that’s a lot …Oh! There we go. Untap. So, you said…Yeah, no, that wasn’t clear.

Participant #110

To help participants better navigate the application, researchers added navigation instructions at the beginning of each section. However, interviewers observed that most participants proceeded to the survey questions without reading the navigation instructions. One participant openly shared his thoughts about the writing prompts.

It was like a lot of writing [haha] not going to lie.

Participant #112

### Intuitive Design of Network Canvas

Overall, all participants responded positively to the application’s design and felt that the application was generally intuitive. One participant expressed that he would be able to use the application on his own without any assistance. The remaining participants expressed that as they moved through the survey, they used the features and tools appropriately.

Once you get the hang of it it’s easy.

Participant #110

Several participants offered recommendations on ways to improve the design of the application. One participant mentioned that a “down arrow” tool be changed to “Next.” He expressed that the proposed tool would make navigating through the application more intuitive.

So maybe like the down arrow—instead of it being a down arrow, maybe it could say “next” or something like that because I’m still looking for the “next” button in the back of my mind. I’m just used to seeing next.

Participant #108

One participant was repeatedly shown how to use many of the tools for the name generator and sociogram features. Although he rarely verbalized his concerns, the interviewer observed that he faced challenges navigating through the application. However, after being shown how to use the tools, he felt confident and found the application easy to navigate. He expressed that people using the application should be taught how to navigate through it.

I believe someone should be there to instruct the person before they use it, and then let them go off on their own after they’ve instructed them on how to use it and, what to do, and how to answer the questions.

Participant #107

## Discussion

### Principal Results

In this study, we used the think-aloud method to assess the feasibility and acceptability of an electronic social network data collection tool, Network Canvas, among BMSM and transgender persons. The think-aloud method was used to assess participants’ cognitive process while completing the social network inventory in Network Canvas. Researchers followed the participants’ cognitive process to understand whether it matched with what was expected of each participant. While in most cases participants’ verbal responses matched their behavior, there were some discrepancies between their verbal affirmation of their use and understanding of the feature versus external observation. Our results suggest that participants were willing to use Network Canvas and found it to be feasible and generally easy to use. However, the sociogram feature and some of the navigation tools required the most instructions for participants. Although participants believed that the design of Network Canvas was easy to understand, they had suggestions for improvement, including more intuitive forward buttons with labels noting the next step. They suggested the inclusion of a brief tutorial before allowing participants to complete the social network inventory on their own. They also noted a need for features and tools to be consistent on each data collection page to improve the application’s intuitiveness.

### Comparison With Prior Work

Instead of interviewer-led assessments, self-report procedures have been utilized in research on vulnerable populations with stigmatized health conditions or behaviors due to their ability to collect valid and reliable measurements of risk behaviors [29,30]. Previous studies have shown that when self-reporting procedures are structured in a way to maximize response accuracy, valid assessments can be collected [31,32]. In this study, navigation prompts were not highly utilized and may not be an effective way to communicate instructions. Participants suggested the implementation of both an orientation prior to using the application, as well as more informative navigation buttons, which are both potential methods to gather more detailed and accurate information in future studies.

The collection of accurate social network information from at-risk populations can be difficult and resource intensive, yet it has the potential to inform targeted interventions with greater impact [33]. Thus, it is crucial to further develop more feasible and efficient methods to collect social network data that can be implemented in a wider range of settings. For example, social network name generation through either paper or interviewer-based methods places a significant cognitive burden on the participant, and it is susceptible to interviewer effects if prompts are asked differently each time [34,35]. This challenge highlights the utility of the Network Canvas tool, which allows for interactive building and complete visualization of the participant’s social network to reduce cognitive burden and is not sensitive to interviewer effects due to the standardized application platform.

Previous research has used the think-aloud method to develop and adapt measurement scales as well as websites and other electronic applications [36]. For example, one study used think-aloud methods to improve the comprehensibility of pediatric antiretroviral therapy adherence measurement items to adapt surveys to cultural context [37]. Another study used think-aloud methods to assess the usability of a smartphone app for the purposes of helping people reduce their alcohol consumption [38]. The findings of this study have been used to further the development of the Network Canvas software. Although interviewer-assisted data collection using Network Canvas is the most optimal, this preliminary data establishes evidence that self-administration of this program is possible, particularly with short, visual tutorials to orient the participant.

### Limitations

This study has a number of important limitations. First, due to the small sample size of this study, we are unable to generalize our findings nor generate greater user feedback data to further improve the usability of Network Canvas. However, during data collection, we reached saturation among participants. Second, there is a risk of reporting bias, in which participants may have given answers in the direction they perceived to be expected by the researchers. Furthermore, participants may have only selected to verbalize the thought processes they wished to share with interviewers (ie, social desirability bias). Third, there is also a risk of acquiescence bias, if participants’ responses tended to have more positive connotations or associations within their stated social networks. Fourth, although we used the think-aloud method to understand how the participant cognitively processed the social network inventory software, this method does require that participants speak aloud throughout the interview, and this was not performed consistently throughout the interview nor across participants, thus likely limiting the observational data we were able to collect [23]. To mitigate the potential effects of this, research assistants were trained to remind and incite verbal feedback from participants, as well as collect observational data that were used to complement participants’ verbatim transcripts during data analysis. Fifth, the functionality of the name generator within the Network Canvas software in which fictitious names were assigned to people in one’s network may limit the generalizability of these results to future studies that may require the use of the real names of contacts in a network. Lastly, although this was part of a larger study focused on assessing BMSM’s willingness to receive sexual health services in pharmacies, the research and data collection protocol was written solely for the purposes of this study.

### Conclusions

Overall, this study showed that Network Canvas is a useful tool to capture social network data, and it has the potential to be a widespread, efficient data collection method. Its ability to be self-administered allowed for participants to provide confidential data about their social networks and their engagements with those social networks. Through observational data, participants asked questions on how to use tools within Network Canvas. Therefore, it would be useful to include short tutorials to enhance participants’ ability to navigate through the application. This study lays the groundwork for further research to assess usability and feasibility in a larger sample of people from different cultural backgrounds who may not be as familiar with the technology. Further evaluation of this self-administered software application to collect social network data has the potential to provide rich descriptive epidemiologic information that can help to guide future prevention strategies.

## Abbreviations

BMSM: Black men who have sex with men
MSM: men who have sex with men
NIDA: National Institute on Drug Abuse
NIMH: National Institute of Mental Health
